# Understanding Cutaneous Mastocytosis: A Case Report on the Symptoms and Diagnosis of a Rare Disease

**DOI:** 10.7759/cureus.89797

**Published:** 2025-08-11

**Authors:** Purva Kulkarni, Prima Selugar, Preeti R Doshi, Parineeta Shelke, Reena Bharadwaj

**Affiliations:** 1 Pathology, Bharati Vidyapeeth (Deemed to be University) Medical College, Pune, IND

**Keywords:** cutaneous mastocytosis, dermis, mast cells, skin disorder, systemic mastocytosis

## Abstract

Cutaneous mastocytosis is a rare skin condition characterized by the accumulation of mast cells, particularly in the dermis, affecting both children and adults. In children, cutaneous mastocytosis is more common, while in adults, it usually progresses to systemic mastocytosis. We present a case report of a three-year-old male child who presented with multiple itchy lesions all over the body for two and a half years. The lesions were spontaneous in nature, reddish in colour, with a wheal-like appearance. The purpose of the case report is to increase awareness and knowledge amongst pathologists about rare disorders.

## Introduction

Cutaneous mastocytosis (CM) is a rare skin disorder characterized by the accumulation of mast cells, especially in the dermis. Systemic mastocytosis (SM) refers to the diffuse infiltration of mast cells in one or more organs, most commonly involving lymphoid tissues, the spleen, and the gastrointestinal (GI) tract [1]. Physiologically, mast cells are distributed throughout the dermis, respiratory tract, GI tract, and genitourinary tract. Mast cells are immune cells involved in inflammatory and allergic responses. They contain granules filled with substances such as histamine, heparin, and cytokines [2]. Mast cells are derived from the myeloid lineage of hematopoietic stem cells and are associated with a range of disorders, including primary and secondary mast cell disorders. Primary mast cell disorders include mastocytosis, mast cell sarcoma (MCS), and mast cell leukaemia, whereas secondary mast cell disorders include allergic and inflammatory conditions. The 2022 WHO classification categorizes mastocytosis into three broad classifications: CM, SM, and MCS [1]. Mastocytosis has a prevalence of approximately one in 10,000 of the population, and the incidence ranges from 5-10 cases per million individuals per year in the United States.

## Case presentation

**Figure 1:**
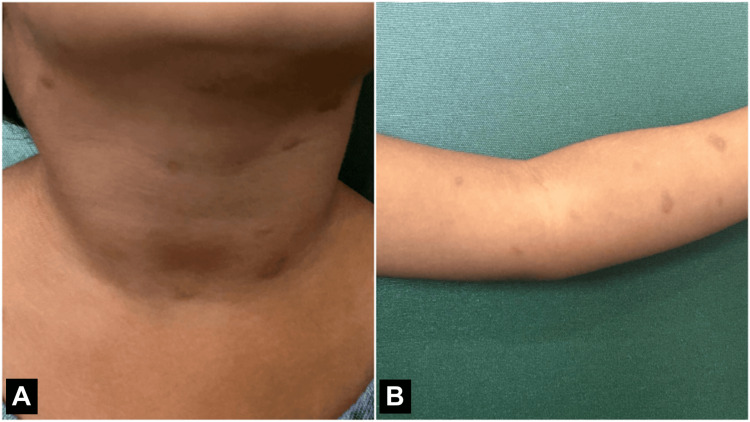
Hyper-pigmented lesions measuring 1.0 x 1.0 cm in the (A) neck and (B) arms

A three-year-old male child presented to the dermatology outpatient department at a tertiary care hospital in September 2024. The child had itchy lesions all over the body since two and a half years. The lesions were spontaneous in nature, reddish in colour with a wheal-like appearance, measuring 1.0 x 1.0 cm (Figure 1), and eventually developed hyperpigmentation. Mild erythema was also noted. Darier's sign was positive. There was no evidence of diarrhoea, abdominal pain, peptic ulcer disease, bone pain, fatigue, or neuropsychiatric changes like mood lability or irritability. There was no evidence of cardiovascular involvement. A 4 mm punch biopsy was sent to the Department of Pathology for histopathological examination. Serum tryptase levels were elevated. Other haematological investigations were within normal limits.

**Figure 2:**
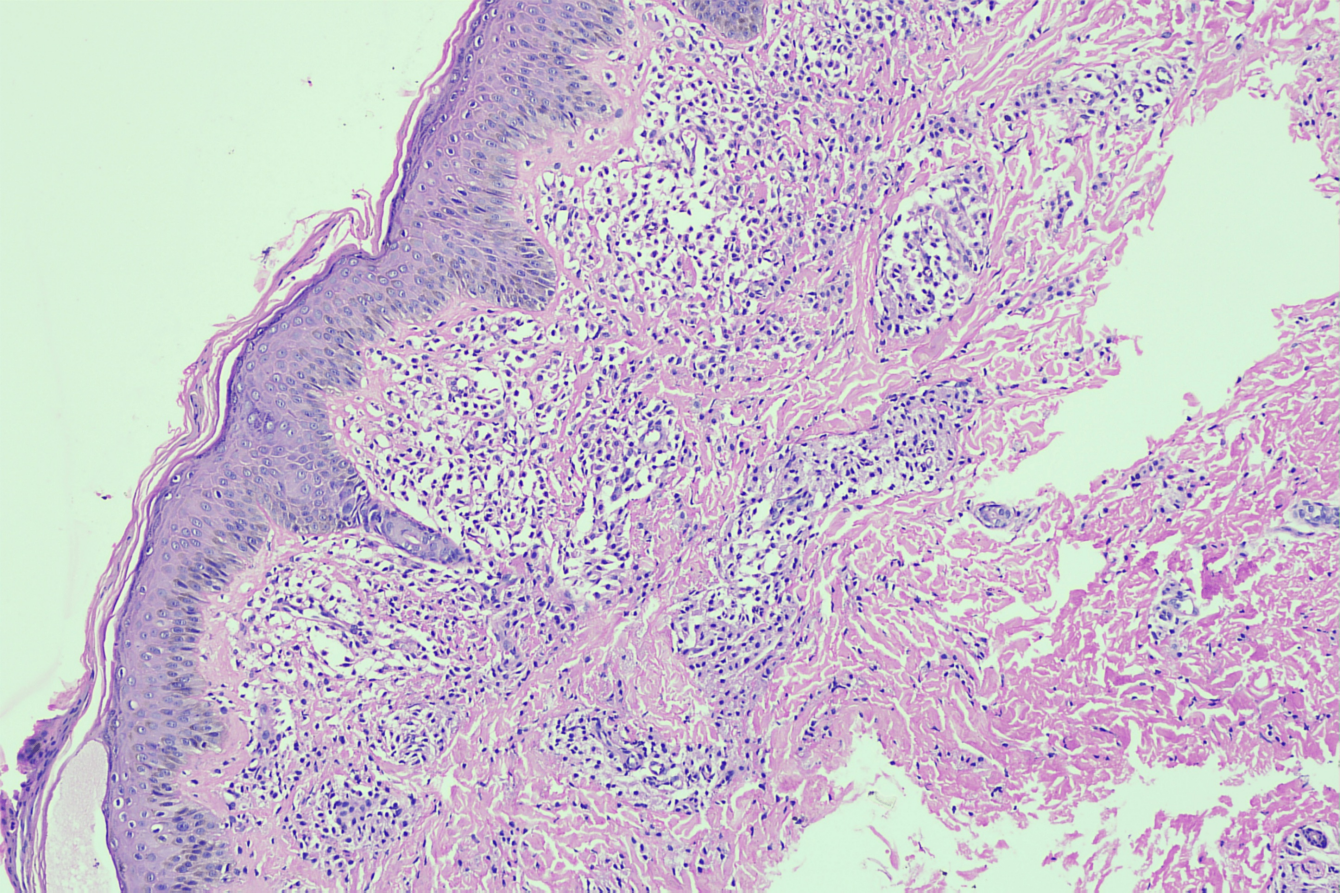
H&E 10× showing the collection of mast cells in the superficial and deep dermis H&E: hematoxylin and eosin

**Figure 3:**
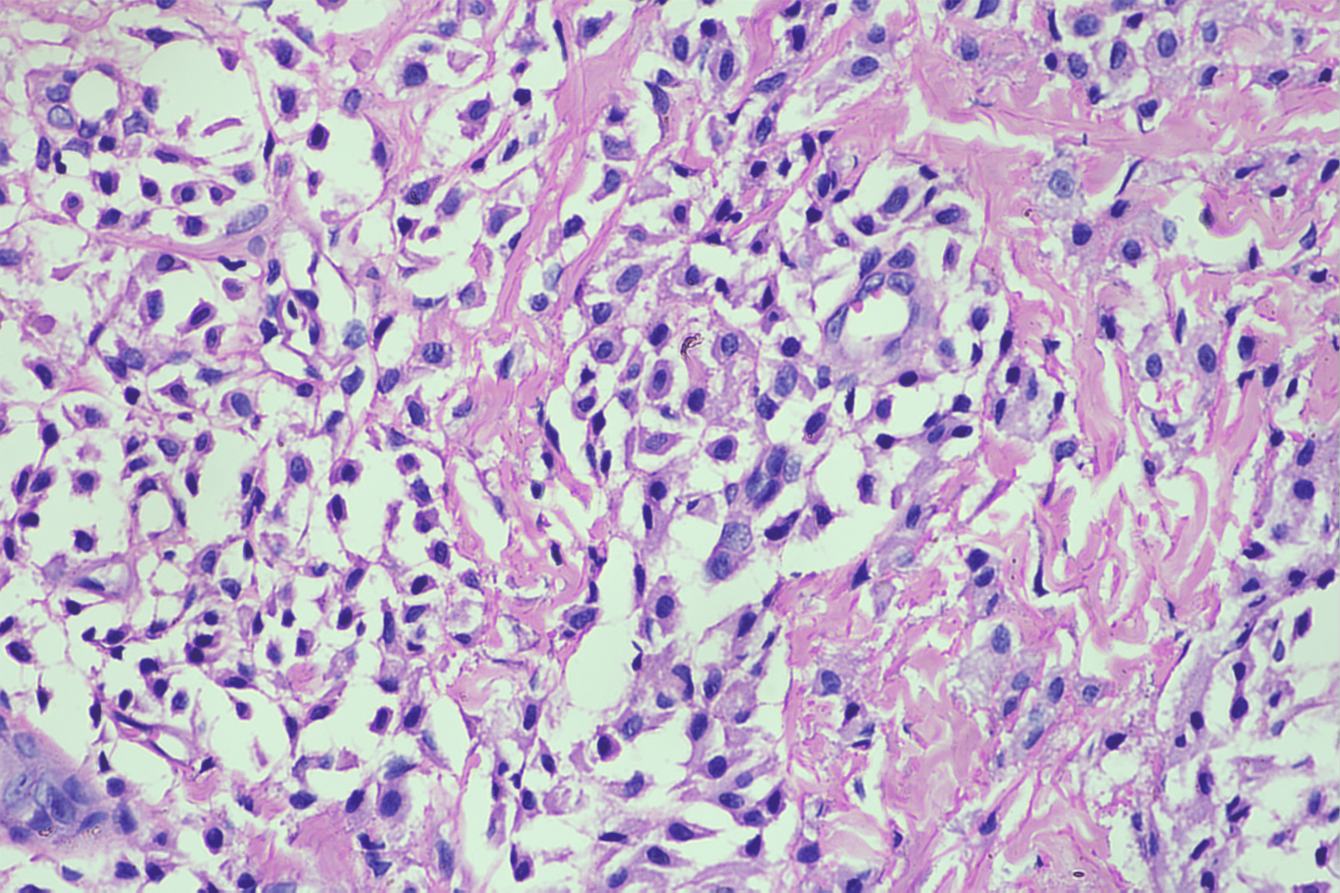
H&E 40× showing round to spindled-shaped mast cells with pale nuclei, abundant granular eosinophilic cytoplasm and distinct cell border H&E: hematoxylin and eosin

On H&E (hematoxylin and eosin) staining, the epidermis showed mild orthokeratosis, and the papillary dermis showed predominantly collections of mast cells (Figure 2). These mast cells were round to spindled in shape with pale nuclei, abundant granular eosinophilic cytoplasm, and distinct cell borders (Figure 3). The reticular dermis was unremarkable.

**Figure 4:**
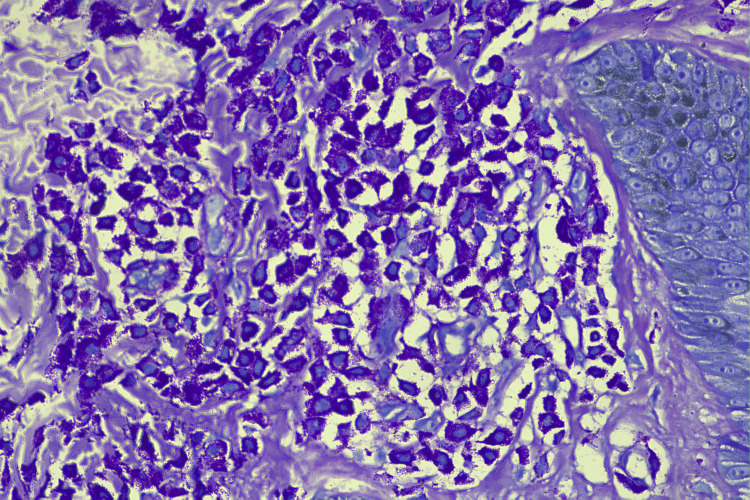
Toluidine blue stain 40×, highlighting the mast cells

**Figure 5:**
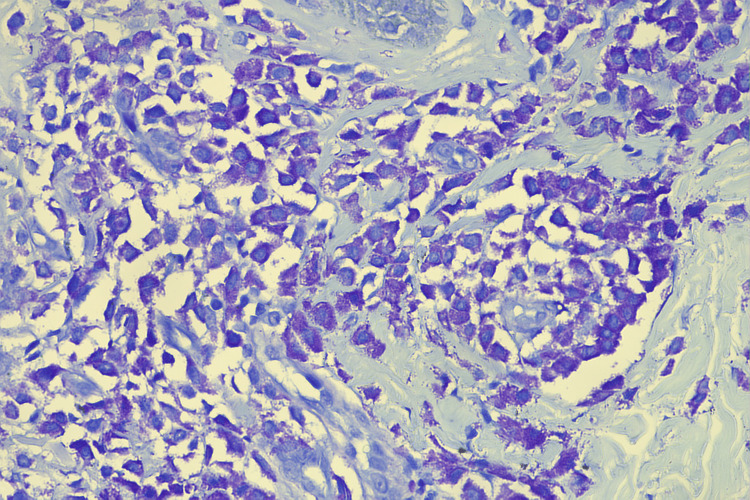
Giemsa stain 40×, highlighting the mast cells

Serum tryptase levels were measured and found to be more elevated than normal at 40 ng/mL (normal range: 1-15 ng/mL). Special stains, like the toluidine blue stain (Figure 4) and the Giemsa stain (Figure 5), were performed.

**Figure 6:**
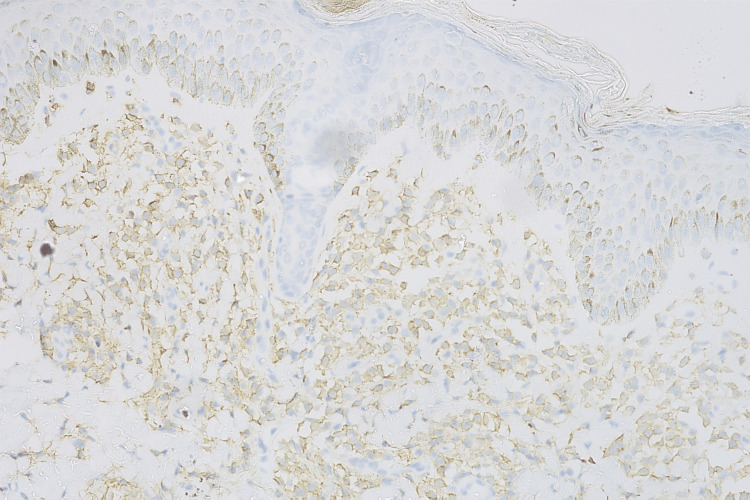
Immunohistochemistry stain for CD117 40×, highlighting mast cells (membranous stain)

Immunohistochemistry for CD117 (Figure 6) was performed, highlighting the granules in the mast cells, which depicted strong positivity for mast cells.

All the features lead to a diagnosis of CM. The dermatologist prescribed topical corticosteroids and antihistamines to the patient. On follow-up, the patient responded well to the treatment, and the lesions were resolved. The patient is being followed up to date, and no new lesions or recurrence have developed after the treatment. There were no referrals. The case was diagnosed, treated, and followed up at a tertiary care centre. Informed consent was obtained for research purposes as well as for treatment.

## Discussion

CM is typically a benign, self-limited disorder in the paediatric population. According to the 2022 WHO classification, CM exists in three forms, of which the most common is maculopapular CM, and all of which may present in children: maculopapular CM (MPCM, also known as urticaria pigmentosa), isolated mastocytoma, and diffuse CM (DCM) [2,3].

The prevalence of mastocytosis has been estimated to be approximately one in 10,000 of the population, with an estimated incidence of 5-10 cases per million individuals per year in the United States. CM occurs more commonly in children (65%) [4]. Adults are more prone to SM, with a double peak in incidence, characterized by an onset at 50 years (indolent course) and 70 years (aggressive course), observed most frequently between 20 and 40 years of age [5].

In mastocytosis, there is abnormal proliferation and accumulation of mast cells, leading to an increased release of these mediators. Physiologically, stem cell factor (SCF) binding to the Kit receptor in the extracellular area activates mast cells. This binding increases the proliferation of mature mast cells, extends their lifespan, and triggers the release of mediators from these cells. In approximately 60% to 80% of mastocytosis cases, somatic mutations of the gene encoding the Kit protein lead to autocrine dysregulation and activation of Kit in the absence of the SCF ligand. The excessive activation of the Kit protein leads to the involvement of internal organs other than the skin. This results in various clinical manifestations, particularly in response to stimuli such as physical triggers (e.g., pressure, temperature), drugs, or stress [6]. It comprises various clinical manifestations, ranging from indolent cutaneous forms to systemic and malignant conditions. The characteristic presentation of mastocytosis consists of cutaneous manifestations: either a solitary mastocytoma, UP, or, less commonly, diffuse CM. Most patients with CM do not meet all the criteria for SM, which is characterized by a massive infiltration of mast cells in various organs, including the bone marrow and liver [7,8]. Although internal organ involvement is rare, CM is often accompanied by GI symptoms and anaphylaxis.

In CM, mast cell accumulation is usually limited to the skin; however, systemic involvement can occur, leading to more severe manifestations, such as GI symptoms (diarrhoea, bloating), cardiovascular symptoms (hypotension), or anaphylaxis. In adults, systemic involvement occurs in about 25% of cases of CM; however, in children, systemic involvement is much less common. The prevalence of systemic involvement increases with age, reaching 15-30% in adults [6].

UP is the most common type of CM [9], marked by reddish-brown spots that itch. Often, Darier's sign is positive in these lesions, indicating that urtication and an erythematous halo are produced in response to rubbing or scratching the lesions. Dermatoscopic features of UP include brown reticular lines, light brown blotches, and a pigment network, which correspond to increased melanocyte growth and melanin production triggered by mast cell growth factors [2].

Isolated mastocytomas are solitary, raised lesions, usually found in infants, especially during the first three months of life. This lesion is often brown or yellowish in colour and can appear as a small bump or nodule. Lesions can be itchy and scratchy, or irritation can cause the mast cells to release histamine, leading to redness and swelling. The lesions are usually harmless and may resolve on their own over time without the need for treatment [2,3].

Diffuse CM is a rare, more severe form of mastocytosis with widespread skin involvement, causing yellow-brown or splotchy skin. It can also lead to systemic symptoms such as hypotension, flushing, and anaphylaxis, and it is more commonly seen in infants [7].

SM affects multiple organs, including the bone marrow, liver, spleen, and lymph nodes. It is further classified into subtypes based on severity, including indolent SM, SM with an associated haematologic neoplasm, aggressive SM, mast cell leukaemia, bone marrow mastocytosis and smouldering SM. Indolent SM is the most common, typically presenting with mild symptoms and a good prognosis [8]. MCS is a very rare, highly aggressive malignancy of mast cells, involving masses or tumours made of mast cells [5].

Differential diagnosis of CM includes melanotic nevi, juvenile xanthogranuloma, urticaria, and Langerhans cell histiocytosis. A detailed clinical history, the appearance of the lesion, a positive Darier's sign, a skin biopsy, and immunostaining help rule out the differentials.

Laboratory investigations, such as serum tryptase levels, should be performed. The elevated serum tryptase is not diagnostic of CM, but it can be useful in supporting the diagnosis. Tryptase is an enzyme released by mast cells during degranulation, and levels may be elevated during acute episodes or systemic involvement. Further blood tests, including a complete blood count, can reveal anaemia, thrombocytopenia, thrombocytosis, leukocytosis, and eosinophilia, which may be present in SM but are less likely to be present in CM. Patients with extensive cutaneous lesions may have elevated urinary histamine excretion, or *KIT* mutation analysis may be warranted [8]. The lesions show a positive Darier's sign, which is pathognomonic of CM.

The management of CM primarily focuses on symptom control, alleviating discomfort, and avoiding triggers that can lead to mast cell activation. Medical treatment includes antihistamines (H1 and H2 antihistamines), topical corticosteroids, and mast cell stabilizers. Avoid triggers like temperature extremes, physical stimuli, and medications such as opiates and nonsteroidal anti-inflammatory drugs (NSAIDs) [8,10].

The prognosis of CM is generally favourable, especially for childhood-onset cases. In many children, CM improves or resolves by adolescence. Not all lesions resolve, but the majority of cases, especially UP and mastocytoma, resolve by adolescence. Adult cases can be more persistent but usually remain confined to the skin, with relatively mild symptoms like erythema [6,9].

Recent studies have provided insights into the genetic mutations (especially mutations in the *KIT* gene) that contribute to the pathogenesis of mastocytosis. Targeted therapies, such as tyrosine kinase inhibitors, are showing promise in treating systemic forms of mastocytosis; however, their role in CM is still under investigation [2,3].

## Conclusions

In a paediatric population, CM is a rare disorder; the incidence ranges from 5 to 10 cases per million individuals per year in the United States. Early diagnosis and appropriate management, including the use of antihistamines and topical corticosteroids, can significantly improve symptoms. Awareness and knowledge amongst consultant dermatologists and pathologists are important for early diagnosis of the disease. Furthermore, research is required to gain a deeper understanding of the disease and its treatment, ultimately improving patient well-being. Serum tryptase levels should be monitored in patients with diffuse CM.
